# Fatigue protocols and athletic performance: a systematic review with a focus on ecological relevance

**DOI:** 10.3389/fphys.2026.1816311

**Published:** 2026-04-22

**Authors:** Ying Yu, Xinbi Zhang, Na Zhang, Yansheng Qu, Xuyang Wang, Ke He

**Affiliations:** 1 School of Physical Education, Shandong University, Jinan, China; 2 School of Kinesiology and Health, Capital University of Physical Education and Sports, Beijing, China; 3 Sports Coaching College, Beijing Sport University, Beijing, China; 4 School of Physical Education, Southwest Jiaotong University, Chengdu, China; 5 Volleyball Management Center of the General Administration of Sport of China, Beijing, China; 6 Sports Department, Beijing University of Aeronautics and Astronautics, Beijing, China

**Keywords:** athletic performance, dual fatigue, ecological relevance, mental fatigue, physical fatigue

## Abstract

**Background:**

Fatigue is a major factor influencing athletic performance, yet findings across studies remain inconsistent. This inconsistency is likely related to substantial heterogeneity in fatigue-induction protocols, fatigue verification methods, and performance outcomes. This systematic review aimed to synthesize current evidence on the effects of sports fatigue on athletic performance and to discuss the ecological relevance of existing experimental fatigue models.

**Methods:**

A systematic search was conducted in PubMed, Web of Science, Embase, and Scopus up to November 2025. Randomized controlled trials with crossover or parallel-group designs investigating athletic performance under fatigue in trained athletes were eligible for inclusion.

**Results:**

A total of 56 experimental studies were included. Physical fatigue generally impaired physical performance, whereas its effects on perceptual-cognitive performance were heterogeneous across protocols, tasks, and outcome measures. Mental fatigue generally impaired endurance, motor skill, and perceptual-cognitive performance, with less consistent evidence for strength- and power-related outcomes. Evidence on dual fatigue was limited but suggested negative effects on endurance and perceptual-cognitive performance.

**Conclusion:**

Current evidence is characterized by considerable heterogeneity in fatigue-induction protocols, fatigue verification methods, and performance outcomes. Single-fatigue paradigms remain valuable for mechanistic isolation, whereas dual-fatigue paradigms may offer complementary ecological relevance by approximating the concurrent physical and cognitive demands of sport. Future research should develop more standardized and ecologically valid fatigue models and further examine potential countermeasures, including neuroenhancement tools and targeted training strategies.

## Highlights

- Findings on fatigue and athletic performance remain inconsistent, partly because of heterogeneity in fatigue-induction protocols, fatigue verification methods, and performance outcomes.
- Single-fatigue paradigms are useful for mechanistic isolation, whereas dual-fatigue paradigms may provide complementary ecological relevance for understanding performance under more realistic sport demands.
- Future studies should prioritize more standardized and ecologically valid fatigue models and evaluate strategies that may help mitigate fatigue-related performance decrements.

## 1 Introduction

Sports fatigue is a complex state involving central and peripheral aspects, directly affecting cardiorespiratory, neuromuscular, physiological, and cognitive functions (Knicker et al., 2011). Sports fatigue, including both physical fatigue and mental fatigue, has been widely studied as a major factor affecting athletic performance. Physical fatigue has long been a major focus of research. Mechanistic studies suggest that it temporarily reduces maximal muscle force and alters central nervous system function (Tanaka and Watanabe, 2011). Studies have shown that physical fatigue can impair physical performance as well as perceptual-cognitive or skill-related outcomes, such as decision-making and tackling technique (Davidow et al., 2020). Noteworthy, after Marcora et al. reported mental fatigue induced by the Stroop task could affect endurance performance (Marcora et al., 2009), drawing attention to the effects of mental fatigue on athletic performance. Increased mental fatigue was observed in world-class players after official matches, with a more pronounced response following a loss (Costa et al., 2023). Mental fatigue caused by prolonged cognitive activity, resulting in an intense sense of fatigue or an inability to maintain energy to accomplish goals (Habay et al., 2021b). Mental fatigue primarily affects central nervous system function and has been associated with reduced endurance, motor skill, and cognitive performance (Kathner et al., 2014; Hopstaken et al., 2015; Guo et al., 2016).

Physical and mental fatigue was reported to be significantly higher after matches than before in team sports players (Ribeiro et al., 2008; Russell et al., 2020; Costa et al., 2023). Understanding the effects of sports fatigue on athletic performance is essential for developing effective strategies to improve resistance and promote recovery. However, some studies have reported that athletes are not affected by fatigue, or may even perform better in decision-making tasks under fatigue (Royal et al., 2006; Parkin et al., 2017). Inconsistencies in research findings may be partly related to differences in fatigue protocols. Athletes experience a combination of physical and mental load (dual fatigue) during training and competition. In laboratory research, physical fatigue and mental fatigue are often induced separately to isolate their specific effects on athletic performance under controlled conditions. This approach is important for clarifying mechanisms and establishing causal inference. However, because athletes often experience concurrent physical and cognitive demands during training and competition, dual-fatigue paradigms may offer additional ecological relevance for understanding performance under more realistic sporting conditions (Mehta and Parasuraman, 2014; Xu et al., 2018).

This review summarizes fatigue-induction protocols and their reported effects on athletic performance. In addition, we discuss why ecologically relevant fatigue models that incorporate both physical and cognitive demands may be useful in experimental research, and we outline potential strategies for performance enhancement under fatigue.

## 2 Methods

This systematic review was conducted in accordance with the Preferred Reporting Items for Systematic Reviews and Meta-Analyses (PRISMA) guidelines (Page et al., 2021). The review protocol was prospectively registered in PROSPERO in April 2025 (registration number: CRD420251003036). A meta-analysis was not performed because of substantial methodological and outcome heterogeneity across the included studies.

### 2.1 Study inclusion and exclusion criteria

Studies were included according to the following participants, intervention, comparator, outcomes, and study design criteria: (1) healthy trained athletes with sport-specific expertise; (2) multiple-joint or whole-body fatigue-induction protocols involving running, jumping, or sport-specific movements; (3) a non-fatigued or control condition; (4) outcomes related to athletic performance; and (5) randomized controlled trials with crossover or parallel-group designs.

The exclusion criteria were: (1) non-athletes, athletes without sport-specific expertise, or injured participants; (2) studies involving additional interventions or confounding factors during the fatigue experiment; (3) single-joint fatigue-induction protocols, such as first dorsal interosseous muscle contractions; (4) non-randomized or uncontrolled study designs; (5) studies reporting only kinematic, kinetic, or biomechanical variables without sport-related performance outcomes; and (6) non-English publications, review articles, conference abstracts, study protocols, and papers without relevant experimental data.

With regard to physical fatigue, single-joint fatigue protocols can help elucidate localized neuromuscular mechanisms, but they are less representative of the whole-body fatigue states typically experienced during training and competition. Because athletes in real sport settings are usually exposed to broader central and peripheral demands, this review focused on multiple-joint and whole-body fatigue protocols.

### 2.2 Search strategy

A systematic search was conducted up to November 2025 in PubMed, Web of Science, Embase, and Scopus. The search strategy combined controlled vocabulary, where available, with free-text terms related to three core concept blocks: (1) athlete/sport, (2) fatigue, and (3) athletic performance. Synonyms within each concept block were combined using OR, and the three concept blocks were combined using AND. Search syntax was adapted to the requirements of each database.

In addition to database searching, the reference lists of all included studies and relevant reviews were manually screened to identify further eligible articles. The full database-specific search strategies, including all keywords, Boolean logic, and database-adapted search strings, are provided in **Supplementary Table 1**.

Literature screening was conducted independently by two reviewers (Y.Y. and X.B.Z.) according to the predefined inclusion and exclusion criteria, and disagreements were resolved through discussion with a third reviewer. Following initial deduplication before screening, a second manual check was performed during full-text assessment to identify any additional duplicate reports or multiple publications arising from the same study.

### 2.3 Screening process and risk of bias assessment

After duplicate removal, titles and abstracts were manually screened independently by two reviewers (Y.Y. and X.B.Z.) according to the predefined inclusion and exclusion criteria. Records were excluded at this stage if they clearly failed one or more eligibility criteria, such as an ineligible population, irrelevant fatigue exposure, absence of an athletic performance outcome, ineligible study design, or non-original publication type. Because multiple exclusion criteria frequently overlapped at the title/abstract screening stage, mutually exclusive reasons were not assigned record-by-record for all excluded records. Detailed reasons for exclusion were recorded only during full-text assessment. No automation tools were used in the screening process.

Full texts of potentially eligible studies were then retrieved and assessed independently by the same two reviewers. Disagreements at any stage were resolved through discussion with a third reviewer. Risk of bias was assessed using the Cochrane risk-of-bias tool, with each domain rated as low, high, or unclear risk (Higgins et al., 2011). Review Manager 5.4.1 software (Cochrane Collaboration, Oxford, UK) was used for the assessment.

### 2.4 Data extraction

Data were independently extracted by two reviewers (Y.Y. and X.B.Z.) using a predefined data extraction form. The following information was extracted from each study: author and year, country, participant characteristics, sport type, fatigue protocol characteristics, control/comparator condition, outcome measures related to athletic performance, and main findings. Any discrepancies in data extraction were resolved through discussion with a third reviewer.

### 2.5 Equity, diversity and inclusion statement

Our authorship team includes both female and male researchers at early-career and senior levels, although all authors are based in one country. We have included data from 29 different countries from Europe, North and South America, Asia, Oceania and the Middle East. Participants included in this review were a mixture of men and women, though some studies reported exclusively on men.

## 3 Results

### 3.1 Search results

**Figure 1:**
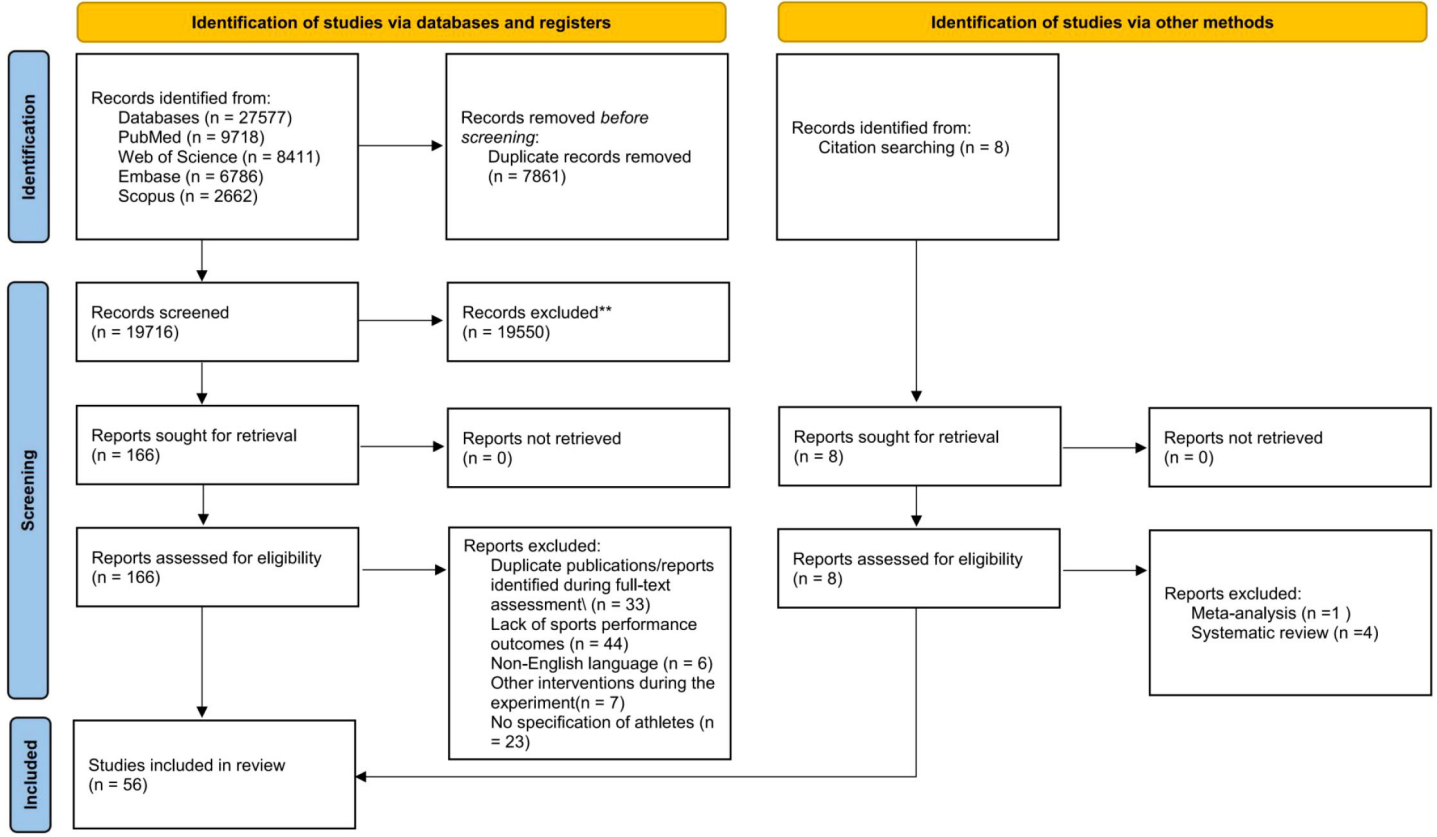
PRISMA flow diagram of study selection. At the title/abstract screening stage, records were excluded when they clearly failed one or more predefined eligibility domains. Because records could meet multiple exclusion criteria simultaneously, mutually exclusive counts were not generated at this stage.

A total of 27,577 records were identified through database searching, including PubMed (n = 9,718), Web of Science (n = 8,411), Embase (n = 6,786), and Scopus (n = 2,662). An additional 8 records were identified through citation searching. After removal of 7,861 duplicate records before screening, 19,716 records remained for title and abstract screening. At this stage, records were excluded when the title or abstract clearly indicated failure to meet one or more predefined eligibility domains, including participant characteristics, fatigue protocol characteristics, outcome relevance, study design, or publication type/language. Because a single record could meet more than one exclusion criterion simultaneously, mutually exclusive counts for individual exclusion reasons were not generated at the title/abstract screening stage. Of the 19,716 records screened, 19,550 were excluded and 166 reports identified through database searching were assessed in full text. Of these 166 full-text reports, 113 were excluded for the following reasons: additional duplicate reports identified after retrieval (n = 33), lack of sports performance outcomes (n = 44), non-English language (n = 6), other interventions during the experiment (n = 7), and no specification of athletes (n = 23). In addition, 8 reports identified through citation searching were assessed for eligibility, of which 5 were excluded because they were meta-analyses (n = 1) or systematic reviews (n = 4). Ultimately, 56 studies were included in the systematic review (**Figure 1**).

### 3.2 Study characteristics

The included studies examined the effects of fatigue on athletic performance across three domains: physical fatigue, mental fatigue, and dual fatigue. An overview of the study characteristics is provided in **Table 1**. In total, the studies covered soccer, rugby, snowboard cross, tennis, table tennis, badminton, handball, Australian football, taekwondo, baseball, futsal, basketball, karate, swimming, volleyball, padel, running, orienteering, and water polo. Regarding fatigue type, 31 studies investigated physical fatigue, 22 investigated mental fatigue, and 3 investigated dual fatigue.

**Table 1:** Fatigue-induced protocols and outcomes on athletic performance.

Author	Participants	Fatigue intervention	Fatigue assessment	Main measurements outcomes
Physical fatigue				
Davidow et al. (2020)	Rugby	8 min prolonged high-intensity intermittent running	–	Tackling technique ↓
Fort-Vanmeerhaeghe et al. (2023)	Team Sport	10 min 30–15 intermittent fitness test	–	Jump performance ↓
Gathercole et al. (2015)	Snowboard cross	60 min low-intensity cycling	–	Jump performance ↓
Bilić et al. (2023)	Tennis	300-m running test	RPE	Change of direction and serve accuracy↓
Cheng et al. (2025)	Badminton	Specific badminton fatigue protocol	RPE	Vertical jumping and agility↓
Torreblanca-Martínez et al. (2020)	Soccer	12 repetitions of 30-m sprint	HR RPE	Kicking accuracy and velocity↓
Loiseau Taupin et al. (2024)	Badminton	8 min specific running test	HR VAS RPE	Gaze behavior during game adapted
Teoldo et al. (2024)	Soccer	90 min specific protocol	RPE	Cognitive performance-
Maly et al. (2018)	Soccer	Yo-Yo IRT1	–	Kicking performance↓
Royal et al. (2006)	Water polo	32 repetitions of water polo-specific drill	HR RPE blood lactate	Decision-making↑, shooting skill performance↓
Jakobsson et al. (2020)	Soccer	Four sets of 4 s cycle ergometer sprints	–	Change of direction↓
Nuño et al. (2016)	Handball	specific handball drills and exercises	RPE	Throwing accuracy and velocity↓
Robineau et al. (2012)	Soccer	90 min soccer game	HR RPE blood lactate	Sprint speed↓, squat jump↓ and vertical jump-
Coventry et al. (2015)	Australian Football	match-specific fatigue protocol	20 m sprint	Punt-kicking speed-
Sant'Ana et al. (2017)	Taekwondo	specific progressive taekwondo test	blood lactate	Kicking performance↓
Freeston et al. (2014)	Baseball	10 min throwing-specific exercise	RPE	Throwing performance↓
Alder et al. (2019)	Badminton	badminton-specific exercise protocol	HR RPE	Anticipation skills↓
Barte et al. (2019)	Soccer	40 repetitions of a 15 m sprint	RPE HR	Passing performance↓
Calderón Pellegrino et al. (2020)	Football	10 repetitions of a 40 m sprint	–	Physical performance in 125↓, 250↑ and 300↑ m^2^ small side games
Dal Pupo et al. (2016)	Futsal	40 min futsal intermittent shuttle-run	–	Sprint performance↓
Armada-Cortés et al. (2022)	Soccer	6 repetitions of a 40 m sprint	–	Hamstring↓, jump performance↓
Ferraz et al. (2012)	Soccer	5 circuit Soccer-specific exercise	HR RPE blood lactate	Kicking velocity ↓in circuit 1 and accuracy-
Ferraz et al. (2012)	Soccer	5 circuit Soccer-specific exercise	HR RPE blood lactate	Kicking velocity ↓ in circuit 1
Ahmed (2013)	Basketball	1 min 70%/90% maximal repetitions chest press and wrist curl	–	Grip Strength↓ and Passing accuracy↓
Yanez et al. (2023)	Futsal	simulated futsal protocol	–	Jump performance↓
Lyons et al. (2006)	Basketball	1 min 70%/90% maximal repetitions squat thrusts	–	Passing accuracy↓
Lyons et al. (2013)	Tennis	Loughborough Intermittent Tennis Test	HR	Groundstroke accuracy↓
Del Percio et al. (2009)	Karate	60 min quadriceps femoris muscle isometric contractions	Blood lactate	Visuo-spatial attention processes-
Thomson et al. (2009)	Team sport	incremental exercise test	VO_2max_	Speed discrimination skills↓
Milioni et al. (2016)	Futsal	40 min simulated game	HR Blood lactate	Finishing kicks performance-
Coutinho et al. (2018)	Soccer	4 min repeated change-of-direction task	–	Behavior during small-sided games ↓
Mental fatigue				
Coutinho et al. (2018)	Soccer	30 min Stroop task	–	Behavior performance in small-sided games↓
Weerakkody et al. (2021)	Australian football	30 min Stroop task	Stroop test performance	Yo-Yo IR1 test↓, goalkicking↓
Staiano et al. (2024)	Team sport	30 min Stroop task	VAS, PVT	Sprint- and jump performance-
Gantois et al. (2020)	Soccer	15/30 min Stroop task	Stroop task performance	Passing decision-making performance↓
Díaz-García et al. (2023)	Padel	30 min Stroop task	VAS, PVT, Stroop task performance	Padel-specific accuracy↓
Fortes et al. (2020)	Soccer	60 min sport-based videogame	VAS	Decision−making skill↓
Penna et al. (2018)	Swimming	30 min Stroop task	VAS	1500-m swimming trial↓
Angius et al. (2022)	Football	30 min Stroop task	Brunel Mood Scale, PVT	Repeated-sprint ability-, psychomotor vigilance↓
Davidow et al. (2020)	Rugby	30 min Stroop task	VAS	Tackling Technique↓
Smith et al. (2016)	Soccer	30 min Stroop task	VAS	Yo-Yo IR1↓, Passing↓, Shooting↓
Soylu et al. (2022)	Soccer	30 min Stroop task	VAS	Technical performance in small-sided game↓
Filipas et al. (2021)	Soccer	30 min Stroop task	VAS	Yo-Yo IR1↓, Passing↓, Shooting↓
Veness et al. (2017)	Cricket	30 min Stroop task	VAS	Cricket-relevant performance↓
Lopes et al. (2020)	Endurance	45 min Stroop task	–	Time-to-exhaustion test↓
Kunrath et al. (2020)	Soccer	30 min Stroop task	–	Performance in small-sided games
Fortes et al. (2024)	Runner	60 min Stroop task/smartphone use	VAS, PVT, Stroop task performance	Jump-, dash performance-
Slimani et al. (2018)	Endurance	30 min Stroop task	VAS	Cognitive and aerobic performance↓
Fortes et al. (2022)	Basketball	60 min sport-based videogame	VAS	Decision-making↓, visual search behavior adapted
Trecroci et al. (2020)	Soccer	30 min Stroop task	VAS RPE	Decision-making in small-sided games↓
Habay et al. (2021a)	Table tennis	60 min Stroop task	VAS	Sport-specific visuomotor task↓
Batista et al. (2021)	Orienteering	30 min Stroop task	PRS MS	Orienteering performance-
Le Mansec et al. (2018)	Table tennis	90 min AX-CPT	VAS	Ball speed ↓, total score ↓, faults ↑, accuracy tended to ↓
Fortes et al. (2021b)	Volleyball	20 sessions 30 min smartphone use	VAS	Decision-making ↓, jump-, endurance-
Dual fatigue				
Alder et al. (2019)	Soccer	30 min soccer-specific drust running with Stroop task	RPE RSME HR	Anticipation↓
Barzegarpoor et al. (2020)	Cycling	65% peak power output cycling to exhaustion with Stroop task	RPE HR	Time to exhaustion↓ MVC↓ PVT↓
Yu et al. (2025)	Volleyball	60 min 60% peak power output cycling with Stroop task	RPE VAS HR	Anticipation -, physical performance↓

HR, heart rate; RPE, rate of perceived exertion; VAS, visual analog scale; PVT, psychomotor vigilance task; ◻, RSME, rating scale of mental effort; MS, motivational state; PRS, Perceived recovery status scale; decreased; -, not affected.

### 3.3 Quality and risk of bias assessment

**Figure 2:**
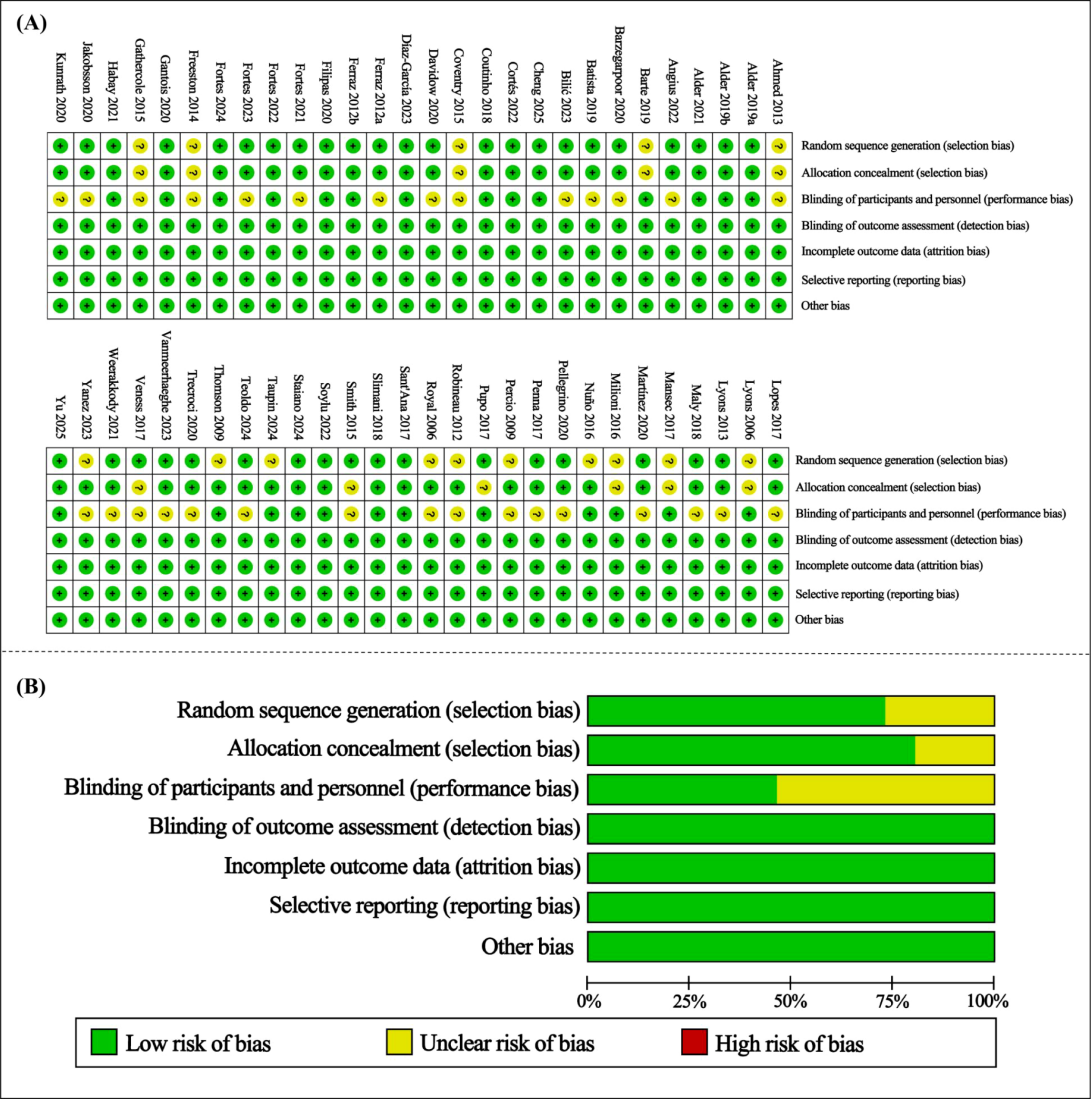
Quality and risk-of-bias assessment. Risk-of-bias summary **(A)** review authors’ judgments about each risk-of-bias item for each included study (red, green, and yellow circles indicate ‘high,’ ‘low,’ and ‘unclear’ risk of bias, respectively); and risk-of-bias graph **(B)** review authors’ judgments about each risk-of-bias item presented as percentages across all included studies. (For interpretation of the references to color in this figure legend, the reader is referred to the web version of this article).

Overall, the included studies showed acceptable methodological quality, and most used controlled experimental designs with a non-fatigued or comparator condition while assessing athletic performance immediately after fatigue induction. However, several domains still showed unclear risk of bias. Eighteen studies exhibited unclear selection bias, mainly because random sequence generation was insufficiently described in 15 studies and allocation concealment was unclear in 11 studies. In addition, 30 studies showed unclear risk for blinding of participants and personnel (see **Figure 2**).

### 3.4 Summary of findings

#### 3.4.1 Fatigue-induced effects on athletic performance

##### 3.4.1.1 Physical fatigue

The whole body needs to be coordinated to accomplish movement, and fatigue in any muscle can significantly affect movement patterns and performance. Physical fatigue primarily decreases neuromuscular capacity, including jump (Gathercole et al., 2015; Fort-Vanmeerhaeghe et al., 2023; Yanez et al., 2023), change of direction (Jakobsson et al., 2020), sprint speed (Robineau et al., 2012; Dal Pupo et al., 2016), and grip strength (Ahmed, 2013). Numerous studies also reported that physical fatigue impaired sport-specific skill performance, e.g. rugby tackling (Davidow et al., 2020), soccer kicking and passing (Maly et al., 2018; Barte et al., 2019), water polo shooting (Royal et al., 2006), handball throwing (Nuño et al., 2016), baseball throwing (Freeston et al., 2014), and taekwondo kicking (Sant'Ana et al., 2017). The decline in physical capacity may partly explain the deterioration in technical performance. However, some studies reported that Australian football punt-kicking (Coventry et al., 2015) and futsal finishing kicks performance were not affected by fatigue (Milioni et al., 2016). Pellegrino et al. reported that physical performance of football players was decreased in 125 m^2^, but improved in 250 and 300 m^2^ small side games (Calderón Pellegrino et al., 2020). The heterogeneous effects of physical fatigue may be partly related to differences in fatigue protocols and the extent of central inhibition they induce.

In addition, the effects of physical fatigue on perceptual-cognitive performance have also attracted widespread attention. It is reported that gaze behavior during badminton game was adapted by physical fatigue (Loiseau Taupin et al., 2024), the accuracy of technical play is affected in tennis (Bilić et al., 2023), soccer (Torreblanca-Martínez et al., 2020), handball (Nuño et al., 2016), and basketball (Ahmed, 2013). Some studies reported that anticipation performance (Alder et al., 2019), speed discrimination skills (Thomson et al., 2009), and behavior during soccer small-side games (Coutinho et al., 2018) was impacted by physical fatigue. However, inconsistent studies were also reported. Del Percio et al. reported that visuo-spatial attention processes in karate players did not change after 60 min fatigue intervention (Del Percio et al., 2009), and cognitive performance in soccer players was not affected by 90 min fatigue protocol (Teoldo et al., 2024). Royal et al. induced physical fatigue by water polo-specific protocol with different rest periods (80s at light level; 40s at moderate level, 20s at high level, and 10s at heavy level), and found better decision-making in high level (Royal et al., 2006). Athletes are often in a particular state of fatigue during training and competition, thus activating more cognitive resources to participate. Tasks used and type of physical fatigue protocols also resulted in inconsistent results (Schapschroer et al., 2016).

##### 3.4.1.2 Mental fatigue

The effects of mental fatigue on athletes have garnered increasing attention, though research findings have been inconsistent. Mental fatigue primarily affects visual search behavior (Fortes et al., 2022) and impairs reaction time during a visuomotor task (Habay et al., 2021a). As a result, endurance (Lopes et al., 2020; Weerakkody et al., 2021), decision-making (Gantois et al., 2020; Fortes et al., 2021b), technique performance (Davidow et al., 2023), and behavior during games (Kunrath et al., 2020) are affected, whereas abilities such as jump, dash performance and repeated-sprint ability (Fortes et al., 2021a; Angius et al., 2022; Staiano et al., 2024) are not. In racket sports, Le Mansec et al. reported that 90 min of AX-CPT-induced mental fatigue impaired table tennis performance, as reflected by reduced ball speed and total score, together with an increased number of faults (Le Mansec et al., 2018). Interestingly, Batista et al. reported that mental fatigue did not affect orienteering performance (Batista et al., 2021). More automated motor patterns may rely less on higher-order cognitive control. This may help explain why mental fatigue does not always impair performance in tasks that involve relatively limited cognitive processing.

##### 3.4.1.3 Dual fatigue

Dual fatigue may more closely reflect real sport settings, in which athletes are exposed to concurrent physical and cognitive demands during training and competition. Alder et al. found that physical and mental fatigue decreased anticipation accuracy, and the decrease was more dramatic in dual fatigue induction (Alder et al., 2021). However, Yu et al. reported that dual fatigue impaired physical performance but not anticipation when compared with physical fatigue alone and a non-fatigued condition (Yu et al., 2025). Barzegarpoor et al. reported that dual fatigue decreased time to exhaustion, psychomotor vigilance task and maximal voluntary contraction in cyclists (Barzegarpoor et al., 2020).

#### 3.4.2 Fatigue-inducing protocols in experimental research

##### 3.4.2.1 Physical fatigue

Physical fatigue-inducing protocols can be classified by form and time. Regarding form, general fatigue protocols included sprinting, cycling, continuous and repetitive upper and lower extremity exercises of varying intensity and repetition (Lyons et al., 2006; Ahmed, 2013; Gathercole et al., 2015; Davidow et al., 2020; Bilić et al., 2023). Depending on the sports characteristics, specific-sport fatigue protocols simulate sports forms by combining different movement speeds or adding specialized movement patterns, such as badminton (Cheng et al., 2025), water polo (Royal et al., 2006), futsal (Yanez et al., 2023), handball (Nuño et al., 2016), Australian football (Coventry et al., 2015), baseball (Freeston et al., 2014), soccer (Ferraz et al., 2012), and some studies used simulated games (Robineau et al., 2012; Milioni et al., 2016). Some studies used related performance test to induce fatigue, such as 30–15 intermittent fitness test (Fort-Vanmeerhaeghe et al., 2023), Yo-Yo IRT1 (Maly et al., 2018), change-of-direction task (Coutinho et al., 2018).

Regarding fatigue-inducing time, short-term fatigue protocols are more time-efficient, most current studies have used different 4–40 repetitions of 15–40 m sprint protocols within 10 min to induce physical fatigue. Long-term fatigue protocols included 40–90 min cycling, quadriceps femoris muscle isometric contractions, and simulated game.

The RPE is commonly used to evaluate physical fatigue. Physical activity can be assessed using HR, blood lactate. Coventry et al. used 20 m sprint speed to evaluate fatigue (Coventry et al., 2015) and VO_2max_ was used in incremental exercise test (Thomson et al., 2009).

##### 3.4.2.2 Mental fatigue

Typically, mental fatigue is induced through cognitive tasks. The Stroop task is the classic protocol to induce mental fatigue, which leads to inhibition of anterior cingulate cortex activation and decreased executive function (Dong et al., 2022). Fortes et al. used sport-based videogame and smartphone usage to stimulate mental fatigue (Fortes et al., 2021b; Fortes et al., 2021a). In general, the mental fatigue intervention time is 30 min, some studies used 15-, 45- and 60-min intervention. In addition to the Stroop task, Le Mansec et al. used a 90-min AX-CPT to induce mental fatigue in table tennis players, further showing that prolonged sustained-attention paradigms can also impair sport-specific performance (Le Mansec et al., 2018).

The visual analog scale (VAS) is the commonly used subjective scale for evaluating mental fatigue, which has high validity in assessing changes in neural excitability under fatigue (Marzouk et al., 2023). Some studies used Brunel Mood Scale, RPE, MS and PRS to assess subjective feeling. Changes in accuracy and reaction time in cognitive tasks were also used in evaluating mental fatigue. Weerakkody et al. used the average reaction time of the first 5 minutes and last 5 minutes in Stroop task as a measure of mental fatigue state (Weerakkody et al., 2021). Some studies used psychomotor vigilance task (PVT) to rate mental fatigue (Angius et al., 2022; Staiano et al., 2024).

##### 3.4.2.3 Dual fatigue

Within the dual-task framework, cycling combined with a Stroop task was the most commonly used protocol (Barzegarpoor et al., 2020; Yu et al., 2025). Alder et al. used 30 min of soccer-specific drust running and completed 25 Stroop trials after 7.5, 15, 22.5, and 30 min of the protocol (Alder et al., 2021). RPE and HR were used to assess physical fatigue, whereas VAS and RSME were used to assess mental fatigue.

## 4 Discussion

The aim of this systematic review was to synthesize the current evidence on the effects of sports fatigue on athletic performance. We identified 56 original studies investigating physical fatigue (31 studies), mental fatigue (22 studies), and dual fatigue (3 studies). Overall, physical fatigue generally impaired physical performance, whereas its effects on perceptual-cognitive performance were inconsistent. Mental fatigue generally impaired endurance, motor skill, and cognitive performance. Evidence on dual fatigue was limited, but available studies suggested negative effects on physical and perceptual-cognitive performance. These between-study differences highlight the translational limitations of current experimental fatigue models.

### 4.1 Existing problems with current research

The current evidence base is dominated by randomized controlled trials that induced physical fatigue or mental fatigue separately. This should not be regarded as a methodological weakness per se. Rather, it reflects a deliberate strategy to maximize internal validity and to isolate the specific effects of different fatigue types on athletic performance. In this sense, single-fatigue paradigms have provided an important mechanistic foundation for the field. However, this also means that the current literature is stronger in mechanistic isolation than in modelling the multidimensional fatigue states often experienced during real training and competition.

**Figure 3:**
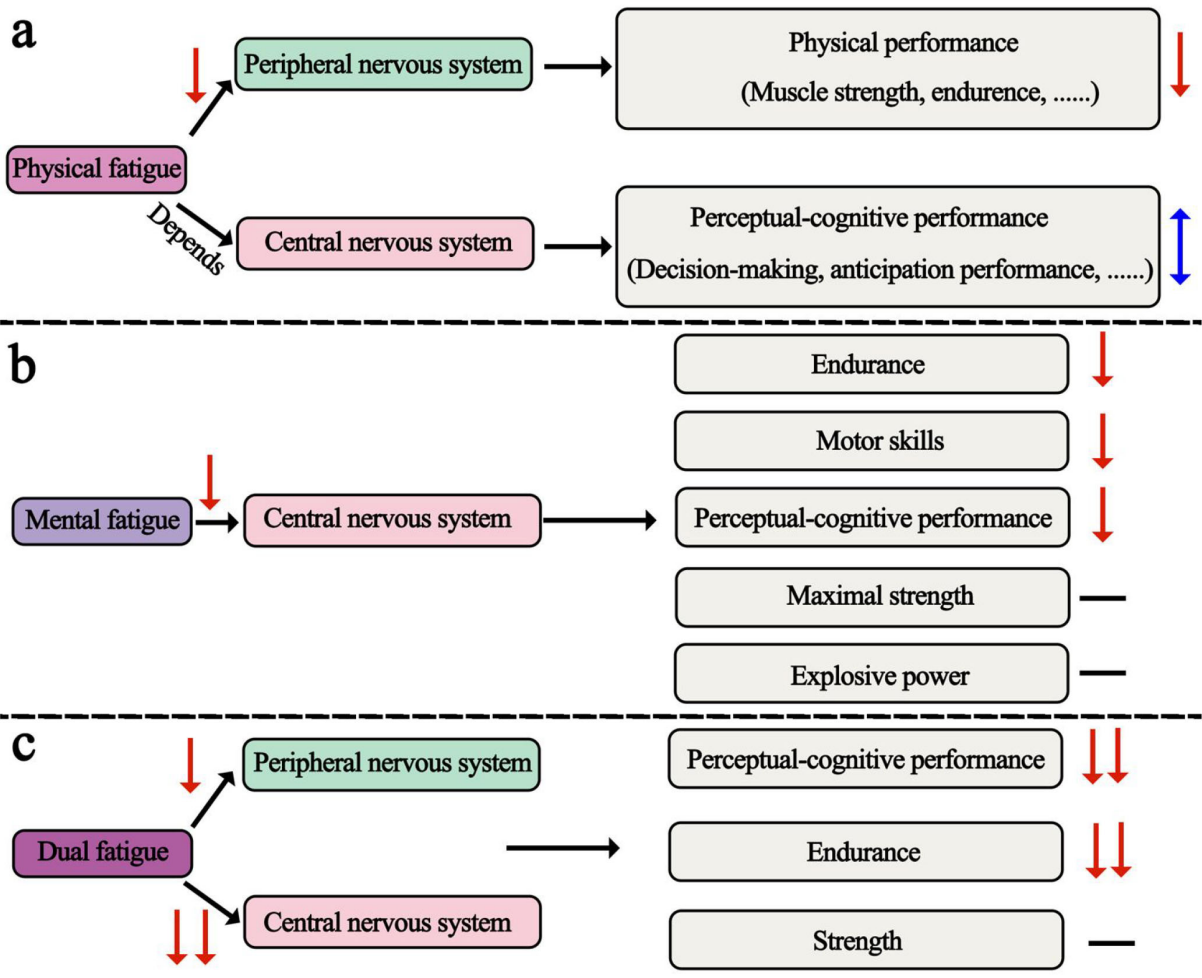
Fatigue-induced effects on athletic performance. **(A)** Physical fatigue. **(B)** Mental fatigue. **(C)** Dual fatigue. ↕, inconsistent results. ◻, decreased, ◻◻, decreased more than physical fatigue, —, not affected.

For physical fatigue, the findings summarized in **Figure 3A** suggest that physical fatigue generally impairs physical performance, whereas its effects on perceptual-cognitive performance are less consistent. One possible explanation is that many existing protocols rely on brief, intense, and standardized exercise tasks, such as repeated sprints or short movement sequences, which often induce fatigue within 10 min. Although these protocols are effective for eliciting acute fatigue, they may primarily reflect short-lasting peripheral impairment rather than the prolonged and integrated fatigue states experienced in open-skill sports. Previous work has suggested that the central effects of physical fatigue are closely related to exercise duration and volume (Thomas et al., 2017). In some studies, physical fatigue was induced within only 4 min (Coutinho et al., 2018), and fatigue induced by four sets of 4 s cycle ergometer sprints has been reported to recover within a few minutes of rest (Jakobsson et al., 2020). Such characteristics may limit the ecological relevance of short-duration protocols for sports such as soccer, basketball, and volleyball, in which athletes are exposed to sustained physical and cognitive demands over much longer periods.

In addition, fatigue-related declines in perceptual-cognitive performance may depend more strongly on central nervous system mechanisms (Menotti et al., 2014). Athletes generally show better cognitive function and greater fatigue resistance than non-athletes, and short-term physical fatigue protocols may even transiently activate the central nervous system in high-level athletes, which may partly explain the inconsistent findings across studies. Long-term and sport-specific fatigue protocols may therefore be more appropriate for investigating the effects of physical fatigue on open-skill athletic performance, as they may better approximate the athlete’s state during training and competition. More importantly, long-term training induces adaptive changes in the brain, and athletes show different patterns of brain activation when performing sport-specific tasks (Olsson and Lundstrom, 2013; Xu et al., 2016). Accordingly, the use of general fatigue-induction methods and non-specific tasks in current studies may contribute to the limited translational value of some findings. Consistent with this view, Schapschröer et al. reported that the effects of physical fatigue on perceptual-cognitive performance are inconsistent and may depend on the specificity of both the fatigue protocol and the perceptual-cognitive task (Schapschroer et al., 2016).

For mental fatigue, the findings summarized in **Figure 3B** indicate generally negative effects on endurance, motor skill, and perceptual-cognitive performance, whereas effects on maximal strength and explosive power appear less consistent. However, several methodological issues remain. First, cognitive tasks lasting longer than 30 min are more likely to induce mental fatigue effectively (Holgado et al., 2020) yet most studies assessed VAS only before and after fatigue induction and rarely reported fixed criteria for confirming a sufficient level of mental fatigue. As a result, it remains unclear whether athletes reached comparable levels of mental fatigue across studies, which may partly explain the inconsistent findings. Second, athletes may have higher cognitive function and greater resistance to the negative effects of mental fatigue than non-athletes (Martin et al., 2016), and the effects of mental fatigue may also differ according to age, sport type, and competitive level (Clemente et al., 2021). Third, although the Stroop task remains the most frequently used paradigm, mental fatigue in sport may arise not only from prolonged cognitive demands but also from emotional strain, competitive pressure, and contextual stressors. Therefore, whether current laboratory-based cognitive tasks adequately reflect the type of mental fatigue experienced by athletes in real sport settings remains an open question.

With regard to dual fatigue, concurrent physical and cognitive demands are common in training and competition, and the available evidence suggests that these fatigue states may interact at the level of the central nervous system (**Figure 3C**). Previous studies have suggested that the superimposition of physical and mental fatigue may accelerate fatigue development and exacerbate central inhibition (Mehta and Parasuraman, 2014; Xu et al., 2018). At the same time, the relative scarcity of dual-fatigue studies in the present review should be interpreted in light of the inclusion of randomized controlled trials, which have largely favored single-fatigue paradigms in order to isolate causal mechanisms under controlled conditions. Therefore, our intention is not to question the value of single-fatigue designs, but to highlight that the current literature provides limited evidence on how physical and mental fatigue may combine under ecologically representative sport conditions. In this context, dual-fatigue paradigms should be viewed as complementary to, rather than replacements for, single-fatigue experimental designs. Mental fatigue decreases brain activation and impairs athletic performance, whereas exercise has been reported to increase cerebral blood flow and brain functional connectivity (Weinstein et al., 2012; Sie et al., 2019), potentially modifying or partially counteracting the inhibitory effects of mental fatigue. This further illustrates both the ecological relevance and the mechanistic complexity of dual-fatigue research.

### 4.2 Ecological relevance of fatigue protocols in experimental research

**Figure 4:**
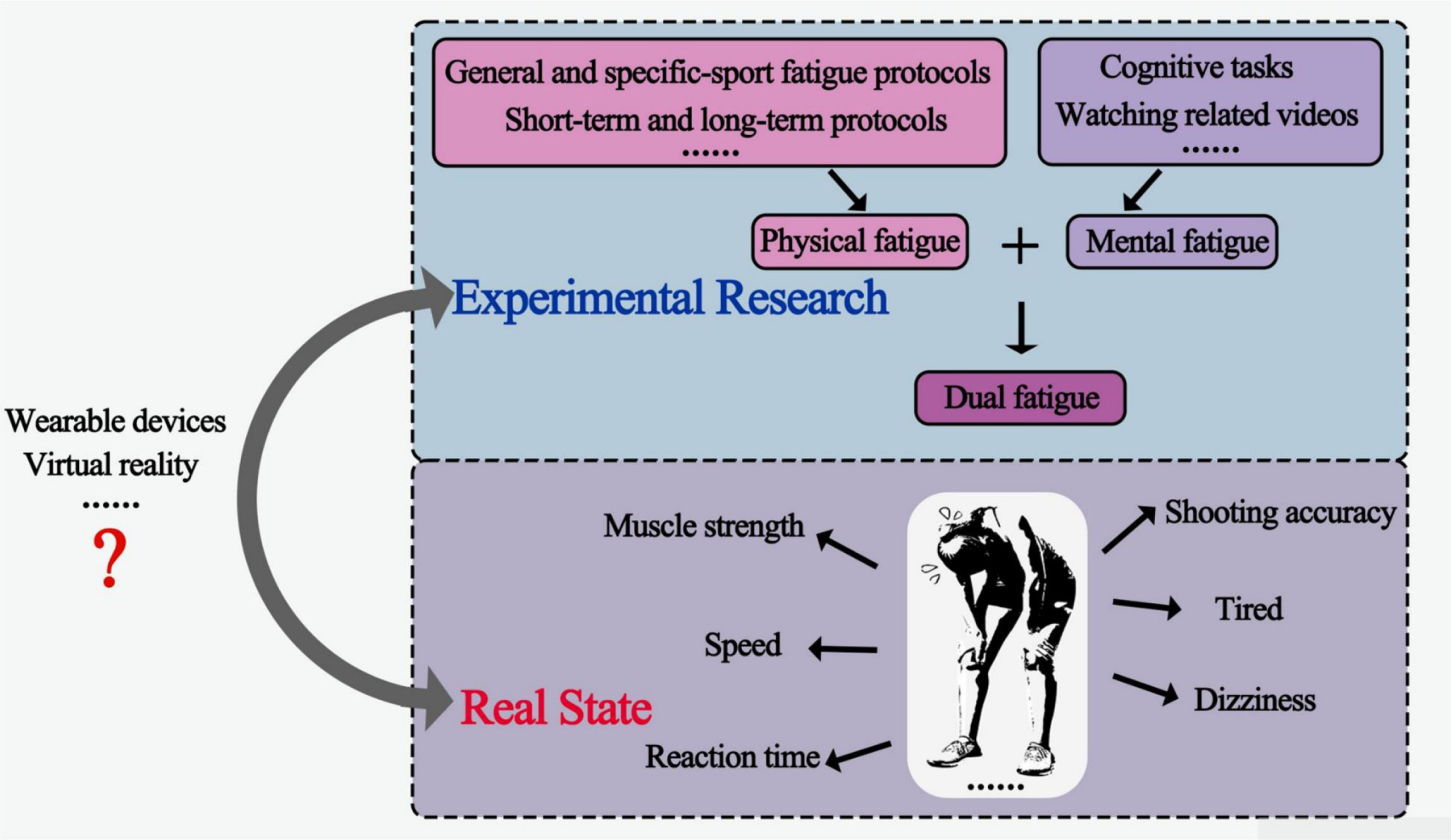
Ecological relevance and translational limitations of current experimental fatigue models in athletic performance research.

Although current research has substantially advanced understanding of fatigue-related changes in athletic performance, further work is needed to improve the ecological relevance of experimental fatigue protocols (**Figure 4**). In our view, dual-fatigue paradigms may complement single-fatigue paradigms by better approximating the concurrent physical and cognitive demands often experienced in real sport settings. However, this potential ecological value must be balanced against reduced internal validity and greater difficulty in causal attribution. For this reason, single-fatigue paradigms remain essential for mechanistic isolation, whereas dual-fatigue paradigms are better considered as ecologically oriented complementary models.

Existing dual-fatigue evidence remains limited, and its interpretation should therefore be cautious. When physical and mental fatigue are induced concurrently, performance changes may reflect the independent effects of physical fatigue, the independent effects of mental fatigue, or their interaction. One type of fatigue may amplify, attenuate, or mask the effects of the other, thereby making mechanistic interpretation more difficult. In addition, inter-individual differences in athletes’ tolerance to physical and cognitive load may increase variability across participants. Therefore, although dual-fatigue paradigms may offer greater ecological relevance, they should not be interpreted as substitutes for controlled single-fatigue experiments.

This complementary perspective is also supported by the limited available evidence. Díaz-García et al. reported that adding a Stroop task to high-intensity interval training increased VAS and rating of perceived exertion, but did not alter heart rate or stroke speed in tennis players (Díaz-García et al., 2023). Likewise, Fuentes-García et al. found that neither physical fatigue nor dual fatigue affected strength manifestations or spirometry in recreational tennis players (Fuentes-García et al., 2021). These findings suggest that dual-fatigue effects may not be uniform across outcomes, tasks, or populations, and further highlight the need for more systematic designs capable of distinguishing isolated and combined fatigue effects.

A staged research framework may therefore be more appropriate. First, single-fatigue experiments should continue to clarify the isolated mechanisms and causal effects of physical fatigue and mental fatigue. Second, factorial designs that treat physical fatigue and mental fatigue as separate factors may provide a stronger bridge between mechanistic clarity and ecological relevance. For example, future studies may compare four conditions: control, physical fatigue only, mental fatigue only, and dual fatigue. Such designs would allow the independent and combined effects of both fatigue types to be examined more explicitly. Third, once these relationships are better understood, more sport-specific or simulated competitive settings may be used to evaluate how combined fatigue states influence athletic performance in practice.

At present, dual fatigue can be induced either sequentially or simultaneously. Sequential paradigms may reflect cumulative or overlapping fatigue loads, whereas simultaneous paradigms may better approximate the concurrent physical and cognitive demands experienced during sport. Cycling combined with a Stroop task is currently the most commonly used simultaneous dual-fatigue model. Although cycling does not fully reflect sport-specific movement patterns, it may simulate a prolonged physiological load while allowing cognitive demand to be standardized across participants. Future studies should further examine whether more representative movement-based and sport-specific dual-fatigue paradigms can improve the ecological validity of experimental fatigue research without excessively compromising mechanistic interpretability.

### 4.3 Performance enhancement under fatigue conditions

In addition, the use of innovative technologies in sport is an expanding area of research. Tools such as wearable devices and virtual reality systems can provide information on movement, speed, distance, heart rate, blood oxygen saturation, autonomic function, and reaction time (Ash et al., 2021), thereby offering useful reference data on fatigue status and physiological characteristics for laboratory-based studies.

Identifying fatigue-induced effects is a prerequisite for enhancing athletic performance under fatigue state. Neuro-enhancement tools such as transcranial direct current stimulation (tDCS) (MaChado et al., 2019) and caffeine (Azevedo et al., 2016) can modulate neural excitability and help counteract the negative fatigue-induced effects. Transcranial direct current stimulation is a non-invasive brain stimulation technique that modulates neural excitability and spontaneous brain activity via low-intensity currents (Paulus, 2000). Salehi et al. reported that tDCS over dorsolateral prefrontal cortex (DLPFC) relieved mental fatigue on 50-meter swimming performance (Salehi et al., 2021). Caffeine has a promoting effect on neural excitability. Due to the mechanism of adenosine, caffeine takes effect 45–60 minutes after intake. However, evidence on caffeine as a countermeasure within sport-specific or ecologically valid fatigue paradigms remains limited.

Brain endurance training builds on the negative effects of mental fatigue on athletic performance by incorporating Stroop tasks into training to increase an athlete’s resistance to mental fatigue. Dallaway et al. found that brain endurance training could improve endurance performance in athletes (Dallaway et al., 2021). Therefore, brain endurance training may decrease the effects of fatigue on neural excitability and thus improve fatigue resistance in athletes.

### 4.4 Limitations

This review was limited to English-language publications, which may have introduced language bias. Although this approach was adopted to maintain consistency in database coverage and screening procedures, relevant studies published in other languages, including Chinese, may have been missed.

## 5 Conclusions

In conclusion, inconsistent findings in the current literature are likely related, at least in part, to heterogeneity in fatigue-induction protocols, fatigue verification methods, and performance outcomes. Single-fatigue paradigms remain important for mechanistic inference, whereas dual-fatigue paradigms may provide complementary ecological relevance for future sport-specific research. However, because evidence on dual fatigue is still limited, conclusions in this area should be interpreted cautiously. Future studies should prioritize more standardized and ecologically valid fatigue models and further evaluate potential countermeasures, including neuroenhancement tools and targeted training strategies.
