# Supplementary material for: Fatigue protocols and athletic performance: a systematic review with a focus on ecological relevance
Source: Front Physiol. 2026 Apr 22;17:1816311. doi: 10.3389/fphys.2026.1816311 (PMC13143685; doi:10.3389/fphys.2026.1816311)
Supplement: Supplementary file 1 [file Table1.docx]

**Supplementary Table S1. Database-specific search strategies**

*Review topic: sports fatigue and athletic performance*

| **Date searched** | From inception to November 2025 | **Databases** | PubMed, Web of Science Core Collection, Embase, and Scopus |
| --- | --- | --- | --- |
| **Manual search** | Reference lists of included studies and relevant reviews | **Core concepts** | Athletes/sport; fatigue; athletic performance |

| **Database** | **Field / platform** | **Search strategy** |
| --- | --- | --- |
| **PubMed** | Title/Abstract + MeSH (where applicable) | (("Athletes"[Mesh] OR "Sports"[Mesh] OR athlete*[Title/Abstract] OR sport*[Title/Abstract] OR player*[Title/Abstract] OR "team sport*"[Title/Abstract] OR "elite athlete*"[Title/Abstract] OR "trained athlete*"[Title/Abstract]) AND ("Fatigue"[Mesh] OR fatigue[Title/Abstract] OR "physical fatigue"[Title/Abstract] OR "mental fatigue"[Title/Abstract] OR "exercise-induced fatigue"[Title/Abstract] OR "neuromuscular fatigue"[Title/Abstract] OR "sport* fatigue"[Title/Abstract]) AND ("Motor Activity"[Mesh] OR "Physical Exertion"[Mesh] OR performance[Title/Abstract] OR "athletic performance"[Title/Abstract] OR "sport-specific performance"[Title/Abstract] OR "physical performance"[Title/Abstract] OR "perceptual-cognitive performance"[Title/Abstract] OR "decision-making"[Title/Abstract] OR skill*[Title/Abstract] OR endurance[Title/Abstract] OR sprint*[Title/Abstract] OR jump*[Title/Abstract])) |
| **Web of Science Core Collection** | TS = Topic | TS=((athlete* OR sport* OR player* OR "team sport*" OR "elite athlete*" OR "trained athlete*") AND (fatigue OR "physical fatigue" OR "mental fatigue" OR "exercise-induced fatigue" OR "neuromuscular fatigue" OR "sport* fatigue") AND (performance OR "athletic performance" OR "sport-specific performance" OR "physical performance" OR "perceptual-cognitive performance" OR "decision-making" OR skill* OR endurance OR sprint* OR jump*)) |
| **Embase** | Title/Abstract/Keyword + Emtree (where applicable) | (('athlete'/exp OR 'sport'/exp OR athlete*:ti,ab,kw OR sport*:ti,ab,kw OR player*:ti,ab,kw OR 'team sport*':ti,ab,kw OR 'elite athlete*':ti,ab,kw OR 'trained athlete*':ti,ab,kw) AND ('fatigue'/exp OR fatigue:ti,ab,kw OR 'physical fatigue':ti,ab,kw OR 'mental fatigue':ti,ab,kw OR 'exercise induced fatigue':ti,ab,kw OR 'neuromuscular fatigue':ti,ab,kw OR 'sport* fatigue':ti,ab,kw) AND ('motor performance'/exp OR 'physical performance':ti,ab,kw OR performance:ti,ab,kw OR 'athletic performance':ti,ab,kw OR 'sport specific performance':ti,ab,kw OR 'perceptual cognitive performance':ti,ab,kw OR 'decision making':ti,ab,kw OR skill*:ti,ab,kw OR endurance:ti,ab,kw OR sprint*:ti,ab,kw OR jump*:ti,ab,kw)) |
| **Scopus** | TITLE-ABS-KEY | TITLE-ABS-KEY((athlete* OR sport* OR player* OR "team sport*" OR "elite athlete*" OR "trained athlete*") AND (fatigue OR "physical fatigue" OR "mental fatigue" OR "exercise-induced fatigue" OR "neuromuscular fatigue" OR "sport* fatigue") AND (performance OR "athletic performance" OR "sport-specific performance" OR "physical performance" OR "perceptual-cognitive performance" OR "decision-making" OR skill* OR endurance OR sprint* OR jump*)) |

**Abbreviations:** Mesh = Medical Subject Headings; Emtree = Embase subject headings; TS = Topic Search; TITLE-ABS-KEY = title, abstract, and keywords.
